# The impact of computerised physician order entry and clinical decision support on pharmacist-physician communication in the hospital setting: A qualitative study

**DOI:** 10.1371/journal.pone.0207450

**Published:** 2018-11-16

**Authors:** Sarah K. Pontefract, Jamie J. Coleman, Hannah K. Vallance, Christine A. Hirsch, Sonal Shah, John F. Marriott, Sabi Redwood

**Affiliations:** 1 School of Pharmacy, College of Medical and Dental Sciences, University of Birmingham, Birmingham, United Kingdom; 2 University Hospitals Birmingham NHS Foundation Trust, Edgbaston, Birmingham, United Kingdom; 3 School of Medicine, College of Medical and Dental Sciences, University of Birmingham, Birmingham, United Kingdom; 4 School of Social and Community Medicine, University of Bristol, Bristol, United Kingdom; University of Brescia, ITALY

## Abstract

**Background:**

The implementation of Computerised Physician Order Entry (CPOE) and Clinical Decision Support (CDS) has been found to have some unintended consequences. The aim of this study is to explore pharmacists and physicians perceptions of their interprofessional communication in the context of the technology and whether electronic messaging and CDS has an impact on this.

**Method:**

This qualitative study was conducted in two acute hospitals: the University Hospitals Birmingham NHS Foundation Trust (UHBFT) and Guy’s and St Thomas’ NHS Foundation Trust (GSTH). UHBFT use an established locally developed CPOE system that can facilitate pharmacist-physician communication with the ability to assign a message directly to an electronic prescription. In contrast, GSTH use a more recently implemented commercial system where such communication is not possible. Focus groups were conducted with pharmacists and physicians of varying grades at both hospitals. Focus group data were transcribed and analysed thematically using deductive and inductive approaches, facilitated by NVivo 10.

**Results:**

Three prominent themes emerged during the study: increased communication load; impaired decision-making; and improved workflow. CPOE and CDS were found to increase the communication load for the pharmacist owing to a reduced ability to amend electronic prescriptions, new types of prescribing errors, and the provision of technical advice relating to the use of the system. Decision-making was found to be affected, owing to the difficulties faced by pharmacists and physicians when trying to determine the context of prescribing decisions and knowledge of the patient. The capability to communicate electronically facilitated a non-interruptive workflow, which was found to be beneficial for staff time, coordination of work and for limiting distractions.

**Conclusion:**

The increased communication load for the pharmacist, and consequent workload for the physician, has the potential to impact on the quality and coordination of care in the hospital setting. The ability to communicate electronically has some benefits, but functions need to be designed to facilitate collaborative working, and for this to be optimised through interprofessional training.

## Introduction

The implementation of Computerised Physician Order Entry (CPOE) has been shown to have many benefits for patients and healthcare professionals, in particular a reduction in medication errors and preventable adverse drug events [1, 2]. Clinical Decision Support (CDS) software is considered the main reason for the observed benefits [3]. However, the introduction of the technology is not without its problems—unintended consequences have the potential to introduce new risks to patient safety and impact on the quality of care [4–6]. As healthcare transactions become more digitised, maintaining effectual communication is a priority for organisations and system developers. Poor or ineffective communication remains one of the leading contributing factors of adverse events in healthcare [7–9] and is repeatedly listed as one of the perceived causes of prescribing errors by those directly involved with such incidents [10–13]. A study conducted in a large acute hospital in the UK found that pharmacist-physician communication via the CPOE system may not be optimal, since a low rate of requests were observed to be actioned, as well as delays in the process [14]. In the study, it was proposed that systems designed to facilitate collaborative communication—such as with bi-directional messaging—may be more effective in practice.

This study aimed to explore pharmacists and physicians perceptions of their interprofessional communication in the context of CPOE and CDS and whether electronic messaging and CDS has an impact on this. The analysis is framed by known topics from both a systemic review of the literature [15] and a quantitative analysis of pharmacist-physician electronic communications via a CPOE system [14].

## Methods

### Ethics approval

This study protocol received favourable opinions and approval by the Research and Development Department at both the University Hospitals Birmingham NHS Foundation Trust (UHBFT) [21st October 2013] and Guys and St Thomas’ NHS Foundation Trust (GSTH) [27th August 2015]. The study was also approved by the University of Birmingham Ethics Committee [ERN_12–0127].

### Methodological approach

Focus groups were selected as the method for gathering the data on the perception of pharmacist-physician communication in the context of CPOE. This approach allowed for data to be generated on the collective views of participants [16] and for opinions and experiences of participants to be shared and contextualised to determine similarities or differences.

### Setting

In the United Kingdom (UK), hospital pharmacists are largely ward-based and work in close proximity to the multidisciplinary team and the patient and their carer/relative. They work collaboratively with medical and nursing staff to ensure patients receive safe and effective treatment(s) that will optimise outcomes [17]. The role of the pharmacist is broad, but in the ward environment primarily encompasses drug history taking, reconciling of medicines (“*the process of identifying an accurate list of a person's current medicines and comparing them with the current list in use*, *recognising any discrepancies and documenting any changes*”[18] and medication review.

Data were collected at two acute hospitals in England: UHBFT and GSTH. UHBFT utilises a locally developed CPOE system, implemented across the hospital since 2003. The system facilitates pharmacist-physician communication with the ability to assign a message (review message) directly to an individual prescription within a patient’s profile [14]. In contrast, GSTH was selected as the CPOE system was implemented on inpatient wards within the preceding 12 months and had no functionality to assign messages directly to individual prescriptions (Table 1). This difference in the two sites is important to help determine whether electronic messaging has an impact on interprofessional communication, or whether other factors, such as the availability of clinical decision support, have an overriding impact.

**Table 1:** Summary of electronic patient records available at UHBFT and GSTH.

Description	UHBFT	GSTH
**CPOE system**	Locally developed PICS	Commercial CareVue (Critical Care) MedChart (In-patient wards)
**Electronic discharge**	PICS	iSoft
**Medical notes**	Paper-based	*ICU*: Electronic and integrated in CareVue *Rest of hospital*: e-Noting separate to MedChart
**Ability to assign an electronic message to a prescription item**	Yes	No
**Other function to communicate with physician**	Nil	Alerts that can appear when the physician generates a prescription

PICS Prescribing Information and Communication System.

### Data collection

Four focus groups were conducted between 2014 and 2015; two uni-professional focus groups and one mixed focus group were conducted at UHBFT, and one mixed at GSTH. At the time of the study in each site, no major changes were made to the CPOE systems. Pharmacists and physicians were eligible to participate in the focus groups if they regularly prescribed or validated inpatient prescriptions within the CPOE system. Participants were invited via email, sent from a member of staff known to the group of professionals. The email provided a background to the research question, dates that the focus group(s) would be held and a copy of the Participant Information Leaflet for further information. All pharmacists working at both hospitals were invited, and a number of physicians were emailed directly, selected based on their likely availability. The eligibility of respondents to participate was confirmed, and the final participants were selected to ensure there was a range of experience with 6–8 participants in each of the groups [16]. Written consent was obtained before the focus group and the discussion audio-recorded. Each focus group was moderated by SP and facilitated by an independent researcher (CH,SS).

### Data analysis

Focus group data were transcribed verbatim and the transcripts uploaded into NVivo 10 to facilitate analysis. A deductive and inductive approach to the analysis was performed. The deductive analysis used a framework of codes (Table 2) identified from the literature [15] and the quantitative analysis of pharmacist-physician communications [14]. This enabled already known concepts to be integrated into the analysis [19], whilst inductive analysis allowed for new or emerging concepts to be identified. The data were initially fine coded to capture detailed descriptions of the data, which were then arranged into the most salient or common themes [20]. Approximately one quarter of the transcribed data were coded by an independent researcher to check for methodological or confirmation bias [21].

**Table 2:** Framework to inform deductive analysis of focus group data.

Origin of theme	Theme
**Systematic review**	A false communication
	Interpersonal communication
	The impact of pharmacist messages in an electronic format
	Physician accessibility to pharmacist alerts
	The effect of CDS on communication
**Quantitative analysis**	Pharmacist assignment of review messages
	Physician action of review messages

## Results

Four focus groups were conducted, three at UHBFT and one at GSTH. A total of 16 pharmacists and 11 physicians participated, with a range of professional experience (Table 3 and S1 Table). The majority of the pharmacists (n = 15/16) and just over half of the physicians (n = 6/11) had experience of paper-based prescribing systems. The mixed focus group at UHBFT had equal participation from pharmacists and physicians, but the mixed group at GSHT had more pharmacists (n = 5) than physicians (n = 2).

**Table 3:** Demographics of focus group participants.

	UHBFT [B]	GSTH [G]	Total
**Pharmacists [P]**			
No. of pharmacists	11	5	16
Experience with paper-based prescribing	10	5	15
Length of time qualified:			
<2 years	2	0	2
2–3 years	3	1	4
Qualified 4–10 years	3	3	6
Qualified >10 years	3	1	4
**Physicians [D]**			
No. of physicians	9	2	11
Experience with paper-based prescribing	4	2	6
Length of time qualified:			
<2 years	5	0	5
2–3 years	1	0	1
4–10 years	2	1	3
>10 years	1	1	2

**Fig 1:**
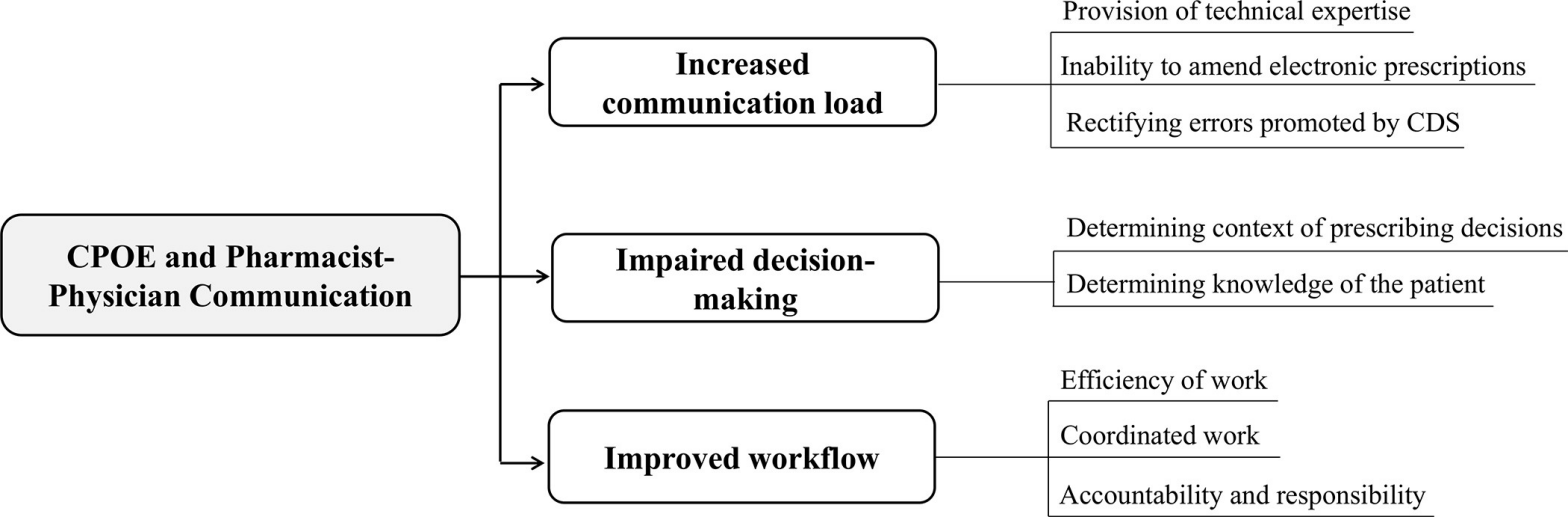
Themes and sub-themes identified in the qualitative analysis of pharmacist-physician communication.

Three prominent themes emerged during the study: increased communication load; impaired decision-making; and improved workflow (Fig 1).

### Increased communication load

#### Provision of technical expertise

The use of a CPOE system introduced a new ‘technical’ expert role for the pharmacist. Pharmacists reported that they were contacted by physicians to find out how to complete complex tasks within the system, such as how to prescribe *“infusions”* [P10.B; D8.B] or complex titration regimens. These requests contributed to *ad hoc* direct and indirect communication between the professionals:

“*That is one of the things that electronic prescribing does introduce*, *which is the technical aspects of knowing how to use the system*. *That’s what we do often get asked*, *“How do I do this” which you never would have had obviously if you were just writing it*” [P15.G].

Pharmacists reported that these requests likely occurred because the training provided to physicians was not optimal, and that they were *“literally thrown in one minute*” [P1.B] and expected to learn about the system on the job and “*pick it up”* [P5.B] over time.

#### Inability to amend prescriptions

In paper-based prescribing environment, pharmacists traditionally annotate prescription orders with information to *“fine tune”* [P9.B] them. Pharmacists reported they had *“a tendency to scribble all over it* [the prescription] *if it was a paper chart to try and make it right*.*”* [P2.B], such as by adding an extra time of day or annotating *“MR”* [modified-release] [P1.B]. Pharmacists across both hospitals said they were unable to fine tune prescriptions within the CPOE system in the same way as they would have done on paper. This led to frustration, since without prescribing rights (i.e. as an independent prescriber), the technology had removed their power to make “*low risk*” [P14.G] amendments that they deemed appropriate. The restriction increased the need to intervene with the physician:

*“I think yes we probably are making more interventions than we would if it was a paper chart*. *Because we’d just write on it rather than making this big thing over it”* [P7.B].

Physicians in both hospitals agreed that pharmacists should be able to amend prescriptions for the benefit of patients, highlighting a trust and confidence in their ability to perform such tasks.

#### Rectifying errors promoted by CDS

The technology was found to increase the frequency with which the pharmacist needed to intervene with the physician. Although it was acknowledged across both sites that CPOE had removed some types of prescribing errors, the technology was found to increase the likelihood of certain error types in the prescribing process that would require pharmacist intervention. Many CPOE systems have the capability to propose order sets when a medicine is selected from a drug dictionary. This decision support provides the prescribing practitioner with the “*full set of information required for a prescription”* [22]. An unintended consequence of these ‘default’ orders was highlighted, since inaccurate prescriptions could be generated through the inadvertent acceptance of the proposed order not intended by the prescriber. Physicians at UHBFT quoted that *“[…] Easily 30–40% of notes* [pharmacist review messages] *are about doses that are different to the standard [CPOE] dose”* [D5.B]. Both professional groups could recount medicines or types of medicines where these errors most likely occurred, such as with “*statins*” [D4.B]. The errors were reported as a particular problem on the admissions wards, where their value for promoting accuracy was questioned:

*“Where the defaults are useful is probably not when you’re taking a drug history because you don’t want somebody to just input the usual dose range*, *you want it to be specific for the patient*.*”* [P10.B]

It did not become clear during the study why order sets were inaccurately selected and generated by physicians, although poor access to medication-related information and the pressure of time were suggested as potential factors.

Errors of ‘selection’ were also reported to occur, particularly with the wrong combination of medicine with a formulation/device such as “*Seretide®*, *the first thing is Accuhaler because it is alphabetical*, *so they’ll just leave it as Accuhaler*” [P5.B].

### Impaired decision-making

#### Determining context of prescribing decisions

Pharmacists and physicians reported difficulties gathering information relating to the context of prescribing decisions that had already taken place. The CPOE systems in both sites were described as effective at providing the information needed to determine what had changed over time—described as a *“massive improvement”* [D1.B] to paper drug charts, which were more difficult because they only lasted a finite period of time (i.e. 2–4 weeks). However, the reasons why prescriptions had changed often prompted a need to intervene with the physician for clarification, either directly or with adding a review message to the prescription. Upon reading a review message from a pharmacist, physicians at UHBFT reported they found it difficult to determine whether a prescription for a patient was generated with intention, or whether errors were actually present. This uncertainty was reported to stem from poor or no documentation of medication-related changes in the medical notes, which had the potential to lead to uninformed changes to prescriptions, and also contribute to delays in actioning requests.

*“By the time they get to the ward*, *it does cross your mind that maybe this prescribing error was on purpose* … *maybe it was changed deliberately in CDU* [Clinical Decision Unit] *in some way*, *and you know you look through the notes and you’ve got no real way of telling*, *so I’ll change it on the assumption that it was mis-prescribed for whatever reason*, *maybe just because the PICS default or something else […]”* [D8.B].

Although some physicians reported that they documented their decision-making and rationale in the medical notes, it was also acknowledged that this was not consistent practice. The use of paper medical notes alongside CPOE, described as a *“half-way position”* [D5.B], was believed to be a contributing factor to this, since the notes were not always present in the workflow when interacting with the CPOE system. As further evidence of this, the documentation of medication-related changes was not raised as an issue by the pharmacists working within the ICU setting at GSTH where both the CPOE and electronic notes are available within the same system.

In an attempt to provide context, some (but not all) physicians at UHBFT adopted a workaround to communicate a rationale for their prescription changes, making them visible to others using an alternative messaging system. Interestingly the workaround to provide the information was consistently reported to occur within the CPOE environment, and not in the medical notes separate to the system, suggesting a preference for all the information to be held in a single place. The strong desire for a *“timeline”* [D2.B] of medication-related changes and facility for *“highlighting anything that’s happened to that drug in the history”* [D5.B] emphasises the importance of an audit trail to access appropriate and relevant information, and the potential for this to have a positive impact on workload.

Pharmacists and physicians at both study sites emphasised the importance of face-to-face communication, and that a “*two-way system*” [D9.B] of communication was more beneficial for discussion. Since pharmacists at GSTH handed over medication-related requests directly, there is more opportunity for discussion to inform decision-making, unlike with the use of uni-directional messaging.

#### Determining knowledge of the patient

Knowledge of the patient was reported an important factor for physicians when making prescribing decisions at the request of a pharmacist. Decision-making was found to be particularly difficult during on-call hours, such as overnight or at weekends. In this situation, physicians were wary about amending prescriptions that were generated by another team, rationalising that it was not their *“duty”* [D2.B] to respond to requests and these were best left to someone who “*might know something more about the patient*” [D3.B].

*“I mean there are occasions when*, *usually ward cover situations*, *where you’re just sort of covering an acute out-of-hour episode and prescriptions relating to their sort of chronic medications*, *I tend to leave them*. *I don’t feel that I am in a position to say ‘why is this amlodipine 5 mg rather than 10* [mg] *when he has been taking 10’*. *There might be a very good reason for it”*. *[D1*.*B]*

Taking responsibility for these requests was difficult given the lack of context regarding medication-related decisions. The pressures of on-call, *“fire-fighting the sick ones”* [D3.B], also provided explanation for why review messages were not prioritised by physicians, except in situations where the acute presentation of a patient was perceived to be medication-related.

### Improved workflow

#### Efficiency

Pharmacists and physicians found that being able to access patient and prescribing information remotely via the CPOE system was beneficial. Time saving was raised as a particular benefit, since both professionals could work remotely to review more patients in a shorter period of time, for example, at weekends. Some pharmacists also used remote working to improve their efficiency, such as to *“[…] collate information*, *look at patients*, *[and] see what needs doing*” [P6.B] prior to attending the ward. However, pharmacists were aware that working remotely could have a negative impact on interactions with patients, relatives of patients and physicians and so chose to avoid this where possible. Physicians at both hospitals reported that pharmacists were visible on the wards.

At UHBFT, pharmacists routinely directed the physician to a specific patient (or bed number) to read their review messages rather than handover the details of their request in person:

*“Yes*, *it’s quite good that you can say ‘go and see beds 9*, *10 and things’ but you don’t have to be specific about every single thing*. . . .*”* [P1.B]

This approach was reported by physicians as being beneficial for their time, rather than being “*stood over*” [D5.B] whilst the changes were made. In contrast, at GSTH where messages could not be documented and assigned to individual prescription orders, each request was communicated and discussed with the physician. The pharmacist would either make these changes with the physician, or follow-up that these have been completed (and completed correctly) if the information were noted down by the physician for action at a later time. Irrespective of the ability to assign a message to a prescription, pharmacists at both sites reported to adopt a workflow that intentionally reduced the number of times they needed to interrupt the physician. They would routinely collate the lower priority tasks to “*pick it all up* [with the physician] *at the end of the day”* [P14.G]. Pharmacists discussed that they did not want to *“pester them constantly”* [P2.B], and at UHBFT assigned review messages to avoid having to *“nag someone about it”* [P3.B]. This demonstrated an awareness of how frequent interruptions may impact on the physicians’ workflow, and how electronic communication could facilitate a reduction in this.

#### Coordinated work

The documentation of review messages at UHBFT facilitated the coordination of care amongst the pharmacists and was used as a means of, “*handing over to other people*” [P2.B] Since the messages are accessible to all users of the CPOE system, pharmacists were reassured that any of their outstanding requests would be followed up by another pharmacist where necessary (e.g. if the patient moved to a different ward). The review message icon on screen made requests visible and accessible, without which “*follow-up would be harder*” [P1.B]. The review message also helped pharmacists identify which patients had been reviewed, facilitating prioritisation of work and avoiding duplication, which would be “*time consuming*” [P5.B]. The ability to assign electronic messages provided benefits beyond simply communicating information to physicians, but also to display activities and actions to coordinate care amongst the pharmacy team.

#### Accountability and responsibility

The ability for pharmacists to document requests via the CPOE systems was perceived to be beneficial for their accountability, particularly compared to interventions made solely through, “*word of mouth*” [P2.B] where documentation of the intervention or interaction may not exist. It was also described as superior to paper notes used in paper-based prescribing processes, where intervention messages may not be filed in the medical notes or go missing:

*“You know you have told them but it’s also documented somewhere for definite that you told them to review something and they can’t say ‘oh you didn’t tell us about this’ so it’s kind of good for us from that communication point of view*, *that we’ve got a trail to say that we did tell them about something*” [P1.B].

The written (typed up) information in a review message was preferred by the physicians, as it was perceived to reduce the risk of errors through misinterpretation or misremembering information that was relayed verbally.

*“There are three drugs they need to change by the end of this ward round and I’ll probably forget one of them or I can’t remember whether she said 15 or 50 [mg]*. *So the readable information is actually very important”* [D1.B].

The review message communication also meant that physicians did not need to rely on their written task lists or handover sheets transcribed from earlier conversations with the pharmacist, which were reported by one physician as, “*notoriously unreliable*” [D5.B] and often, “*adulterated by other clinicians*” [D5.B]. The documentation of the review messages was therefore also used as a, *“safety net*” [P1.B] by pharmacists to document information or to back-up information relayed verbally to the physician and to provide more detailed information.

## Discussion

This study aimed to investigate the impact of CPOE on pharmacists-physician communication in the hospital setting, and whether electronic messaging and CDS has an impact on this. Three prominent themes emerged during the research, showing CPOE to have an impact on the frequency of communications between the professionals, decision-making and workflow.

### Communication load

Physicians were found to rely on pharmacists to provide technical expertise when they needed assistance with medication-related tasks within the hospital CPOE systems. This was found to continue in an environment with a well-established CPOE system in use. Pharmacists associated the increased workload from technical queries to gaps in the physicians’ knowledge of the system and the limited time allocated to training. The informal role of technical expert has previously been described by McMullen *et al* (2015), who found that pharmacists became “*informal trainers*” of systems post-implementation of CPOE and spent time showing physicians how to efficiently use it [23]. However, in contrast to the findings in this study, McMullen *et al* (2015) also found that the support demanded from physicians *“diminished with time”* and experience. The continued demand for informal education from the pharmacists suggests that physicians find it beneficial and that pharmacists are generally well-placed and accessible to perform the task. This *ad hoc* guidance is likely to fall over a weekend though, since ward-based pharmacy services at the weekend are still uncommon in hospitals in England [24]. Socio-technical incidents at UHBFT have previously been found to occur more frequently on a Sunday compared to the rest of the week (p<0.013) [4], which may reflect a lack of informal training and support at this time. Training has been identified as a key consideration for successful implementation and on-going use of CPOE [25, 26]. Insufficient training can lead to sub-optimal use of systems—the use of the technology in a way that is not intended (i.e. workarounds) or underuse of system functions—which may increase the risk of error [4, 27, 28]. This was perceived to be a contributing factor for the sub-optimal use of some system functions at UHBFT, such as with the ‘sign-off’ function to indicate that a review message had been acknowledged. Cresswell *et al* (2013) recommend that “*the most effective training is tailored to the individual roles of users*, *without being too restrictive as this can undermine understanding of how the whole system functions*”. Although this may be true in ensuring routine tasks can be completed to deliver everyday care, it may not consider the use of the system in relation to interprofessional communication or how best to use system functions to coordinate care. Such knowledge of the system may only really be gained through interprofessional training, so that practitioners can develop skills together in the context of CPOE [29].

CPOE systems can enforce or reinforce professional standards and boundaries [30], for example, by restricting actions according to profession or grade. The reduced ability to amend or “*fine tune*” prescriptions, compared to the freedom had on paper charts, was found to increase the communication load for the pharmacist. Pharmacists’ previously written endorsements on paper drug charts have been found to “*subtly influence medical prescribing*”[31] and are conducted with the intention to benefit patient care. Previously made known to the physician by a different coloured pen, or an allocated space on the chart, the pharmacist would amend low risk errors that they felt competent to action. In the context of CPOE, this was not always possible, and instead required handover of the task to the physician. The change in communication load highlights the importance for systems to be flexible and designed to account for existing work processes (i.e. in a paper-based environment). However, the very fact that systems have not allowed pharmacists to make changes may cast doubt on whether pharmacists were previously acting outside the scope of their practice when working in paper-based processes. Standards clearly state that pharmacists should “*intervene with prescribers*”[32] to ensure the safe and effective use of medicines. Further clarification may be required from professional bodies as to the extent to which prescription orders in a CPOE system can be amended by non-prescribing pharmacists. Amendments made to paper drug charts have been found to influence prescribing [31], and the potential for learning to be gained from these should not be overlooked. In view of this, where amendments are possible, these should also be clearly visible to other practitioners (e.g. in colour).

Pharmacists and physicians in this study were aware of new error types relating to the use of the technology. CDS in the form of default orders (sometimes referred to as auto-complete or auto-populated orders) and drop-down menus can lead to prescribing errors through the acceptance or selection of an incorrect order [5, 33]. The occurrence of these errors at the study sites was found to add to the communication load of the pharmacist, and therefore the workload of the physician. The use of default orders to *“nudge”* practitioners along an appropriate course have been shown to be effective at instilling and maintaining a required standard of prescribing [34]. However, nudging towards a regimen that has the potential to vary depending on the patient and/or the indication for treatment may be less beneficial in practice. Acceptance of incorrect orders may suggest that physicians are not only interacting with the system quickly, but also with automatic unconscious thinking—so called “*System* 1” thinking—where less attention is paid to the detail of the order and little or no effort is applied to the review [35]. Kahneman (2011) describes how some activities can become “*automatic through prolonged practice”* and that System 1 thinking has learned associations. Orders may be accepted as correct through association (i.e. of the most common regimen), without conscious thinking to check that the regimen is consistent with the patient’s needs—omitting an *“attentional check*” [36] to ensure the populated prescription on screen matches the patient’s medication history. The use of default orders in CPOE systems may have the unintended effect of encouraging this System 1 thinking when generating a prescription, leading to an over-reliance on the CPOE system to make decisions, and active failures (as slips) to occur [36]. As such, the use or design of default order sets in systems requires further investigation, particularly for regimens that can vary between patients.

### Decision-making

CPOE systems at the study sites were found to facilitate access to information to determine what treatments had changed for patients over time, but the reasons why remained largely unknown. Systems did not provide the capability to document a rationale, and for this to be accessed retrospectively. On receipt of a request from the pharmacist, physicians reported that they would often struggle to make decisions, since a lack of documentation and poor access to information made it difficult to determine whether the prescription was as intended, or whether there was an unintended discrepancy. The use of electronic patient records has previously been found to impact on physicians’ clinical reasoning, because although systems can provide a lot of patient data, it is not always easy to gain enough knowledge of the patient to inform decision-making [37]. In this study, physicians expressed a need for a function within the CPOE system to provide a *“timeline”* of events, so that prescriptions changes over time could be viewed to ascertain the patient’s treatment journey. This is consistent with a study that found clinicians want to *“build the patient story”* [38] when delivering care, and reported that the electronic patient record presents fragmented information, which make the story difficult to construct. A function within CPOE systems to facilitate this would be beneficial and would help avoid uncertainty during the medication process. Uni-directional messages within systems also means that physicians are unable to provide a rationale for their decision-making. Incorporating bi-directional communication into CPOE systems could enhance the effective flow of information, which in turn can help manage workload and enhance coordinated care [39, 40].

### Workflow

Pharmacists in both hospitals favoured a workflow that minimised interruptions for the physician, prioritising the workflow of the physician over the need to remove tasks from their own workload and working memory. This *“appropriate obtrusiveness”* [41] was found to be in contrast to some studies that show hospital work to be more task driven and dependent on interruptive communication so that tasks can be completed in a timely manner [42–44]. The capability to communicate requests electronically—independently of the location and activity of the physician—facilitated a non-interruptive workflow and also meant that the length of any interruption could be reduced. This may be favourable since interruptions unrelated to the task at hand can lead to multi-tasking for the physician [43], which can impact on their working memory [42, 45] and shorten overall time spent on tasks [46, 47]. The non-interruptive workflow routinely adopted by pharmacists suggests that they are socially aware and sensitive to the activities or tasks being carried out by physicians [48, 49]. This awareness is possible when working in close proximity to other healthcare professionals (such as on the ward). It is important to note that although the desire to minimise the length of interruption is conducted with the best intentions, it does have the potential to reduce opportunities for informal interaction and formal discussion [50], both of which are essential to gain context and to promote collaborative working practice [51, 52].

The ability to assign messages to prescriptions was found to facilitate the coordination of work between pharmacists and reduced the risk of information being misremembered or misinterpreted by the physician. Unlike requests that are discussed or handed over verbally, the assignment of a message generates data within the CPOE system which is recorded in the patient’s clinical record. This was perceived to be useful for accountability, since it could be used as evidence that a task had been communicated by the pharmacist, which in turn could be used for organisational audit and monitoring.

### Strengths and limitations

The four focus groups were conducted in only two hospital sites in England. Although the salient themes emerged across both settings, which may provide some evidence of data saturation, the findings should be interpreted in the context of the settings investigated, and may not be transferable to all hospitals, or settings outside the UK. This is particularly the case with data generated at the comparator site, where only one focus group was conducted and so data saturation is less likely to have been achieved. Both uni-professional and mixed focus groups were conducted at UHBFT, and one mixed focus group at GSTH. The approach taken at GSHT could mean that participants of the same profession were less forthcoming with their shared experiences, to avoid expressing opinions that may offend or initiate debate with the other professional group. However, the mixed group in this setting did allow participants to challenge each other’s views relating to the barriers and facilitators to their interprofessional communication. The participants in each focus group had a range of professional experience. However, owing to the number of participants overall, the results may not be representative of the entire population of pharmacists and physicians working in the two hospitals. Steps were taken to reduce the risk of methodological or confirmation bias during the investigation and to gain a range of perspectives.

## Conclusions

The capability to communicate electronically facilitated a non-interruptive workflow, beneficial for staff time and for limiting distractions. It also improved clinical documentation, and facilitated the coordination of care. However, the use of CPOE was found to increase the frequency of communications between pharmacists and physicians, owing to insufficient knowledge of how to use the systems, system restrictions, and errors potentially generated by decision support software. Decision-making was also found to be affected owing to the difficulties faced when gathering and contextualising patient information from the system. These factors need to be considered in the design of systems, and supported by interprofessional training to optimise communication between professionals.

## Supporting information

S1 Table Detailed demographics of focus group participants. (PDF)
