# Supplementary material for: The impact of computerised physician order entry and clinical decision support on pharmacist-physician communication in the hospital setting: A qualitative study
Source: PLoS One. 2018 Nov 16;13(11):e0207450. doi: 10.1371/journal.pone.0207450 (PMC6239308; doi:10.1371/journal.pone.0207450)
Supplement: S1 Table — (PDF) [file pone.0207450.s001.pdf]

S1 Table. Detailed demographics of focus group participants.

Setting	Focus group	Identifier	Profession	Setting	Gender	Length of time qualified	Experience with paper prescribing
**UHB**	Uni-professional (pharmacists)	P1.B	Pharmacist	UHB	F	4–10 years	Yes
		P2.B	Pharmacist	UHB	F	2–3 years	Yes
		P3.B	Pharmacist	UHB	M	2–3 years	Yes
		P4.B	Pharmacist	UHB	F	2–3 years	Yes
		P5.B	Pharmacist	UHB	F	<2 years	No
		P6.B	Pharmacist	UHB	F	<2 years	Yes
		P7.B	Pharmacist	UHB	F	4–10 years	Yes
**UHB**	Uni-professional (physicians)	D1.B	Physician	UHB	M	4–10 years	Yes
		D2.B	Physician	UHB	M	< 2 years	No
		D3.B	Physician	UHB	F	<2 years	No
		D4.B	Physician	UHB	M	<2 years	No
		D5.B	Physician	UHB	M	<2 years	No
**UHB**	Mixed	P8.B	Pharmacist	UHB	F	<2 years	Yes
		P9.B	Pharmacist	UHB	F	>10 years	Yes
		P10.B	Pharmacist	UHB	F	>10 years	Yes
		P11.B	Pharmacist	UHB	M	4–10 years	Yes
		D6.B	Physician	UHB	F	4–10 years	Yes
		D7.B	Physician	UHB	M	>10 years	Yes
		D8.B	Physician	UHB	M	<2 years	No
		D9.B	Physician	UHB	F	2–3 years	Yes
**GSHT**	Mixed	P12.G	Pharmacist	GSTH	F	>10 years	Yes
		P13.G	Pharmacist	GSTH	F	4–10 years	Yes
		P14.G	Pharmacist	GSTH	F	2–3 years	Yes
		P15.G	Pharmacist	GSTH	F	4–10 years	Yes
		P16.G	Pharmacist	GSTH	M	4–10 years	Yes
		D10.G	Physician	GSTH	M	>10 years	Yes
		D11.G	Physician	GSTH	M	4–10 years	Yes

F Female; M Male
